# Supplementary material for: β‐catenin signaling modulates the tempo of dendritic growth of adult‐born hippocampal neurons
Source: EMBO J. 2020 Sep 15;39(21):e104472. doi: 10.15252/embj.2020104472 (PMC7604596; doi:10.15252/embj.2020104472)
Supplement: Supplementary file 1 — Appendix [file EMBJ-39-e104472-s001.pdf]

Appendix to:

β-catenin signaling modulates the tempo of dendritic growth of adult-born hippocampal neurons

Jana Heppt[1], Marie-Theres Wittmann[1,2], Iris Schäffner[1], Charlotte Billmann[1], Jingzhong Zhang[3,4], Daniela Vogt-Weisenhorn[3], Nilima Prakash[3,5], Wolfgang Wurst[3], Makoto Mark Taketo[6], D. Chichung Lie[1#]

content:

Appendix Figure S1: Potential regulators of canonical Wnt signaling during adult neurogenesis

A

Wnt/β-catenin signaling

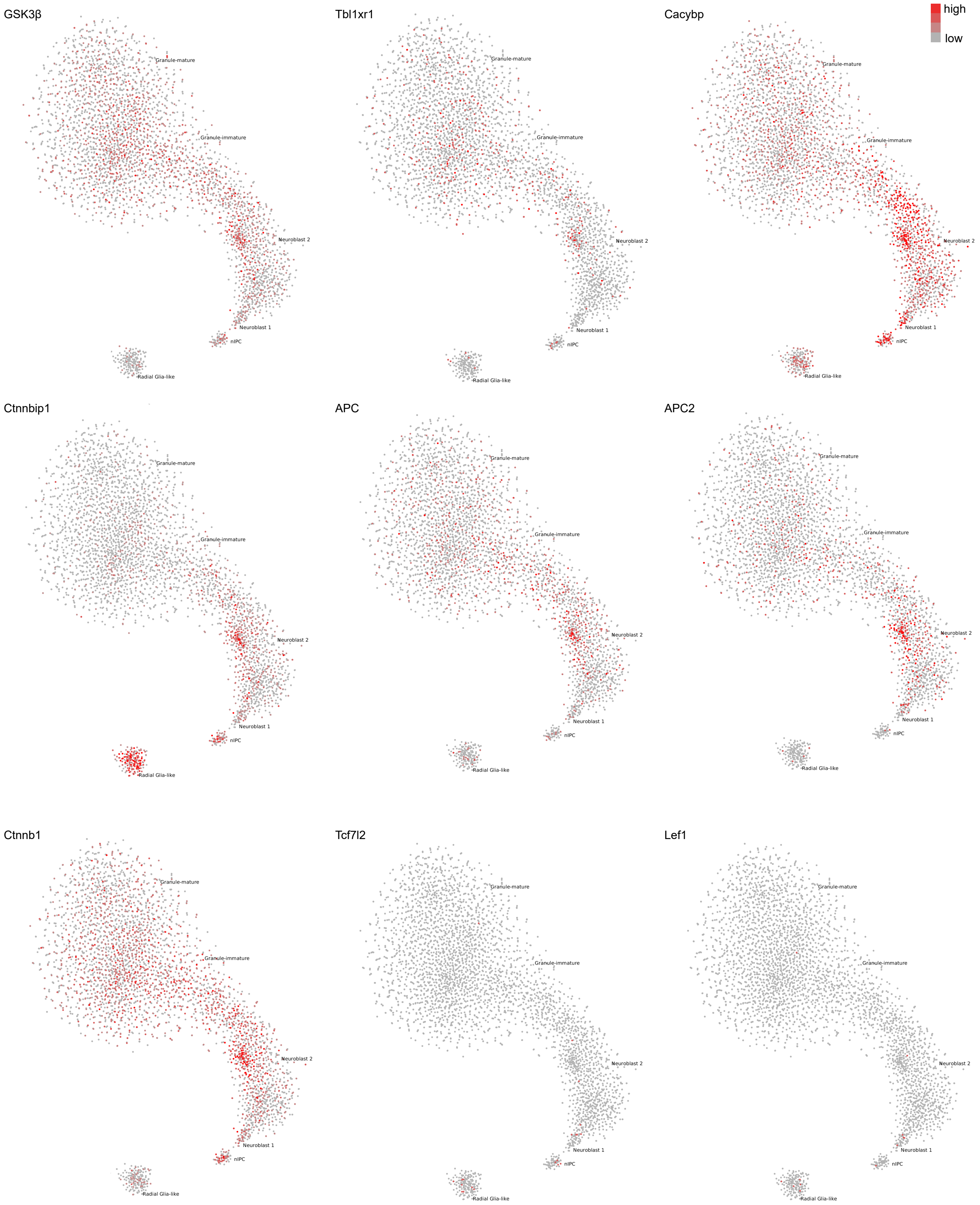

Wnt/PCP signaling

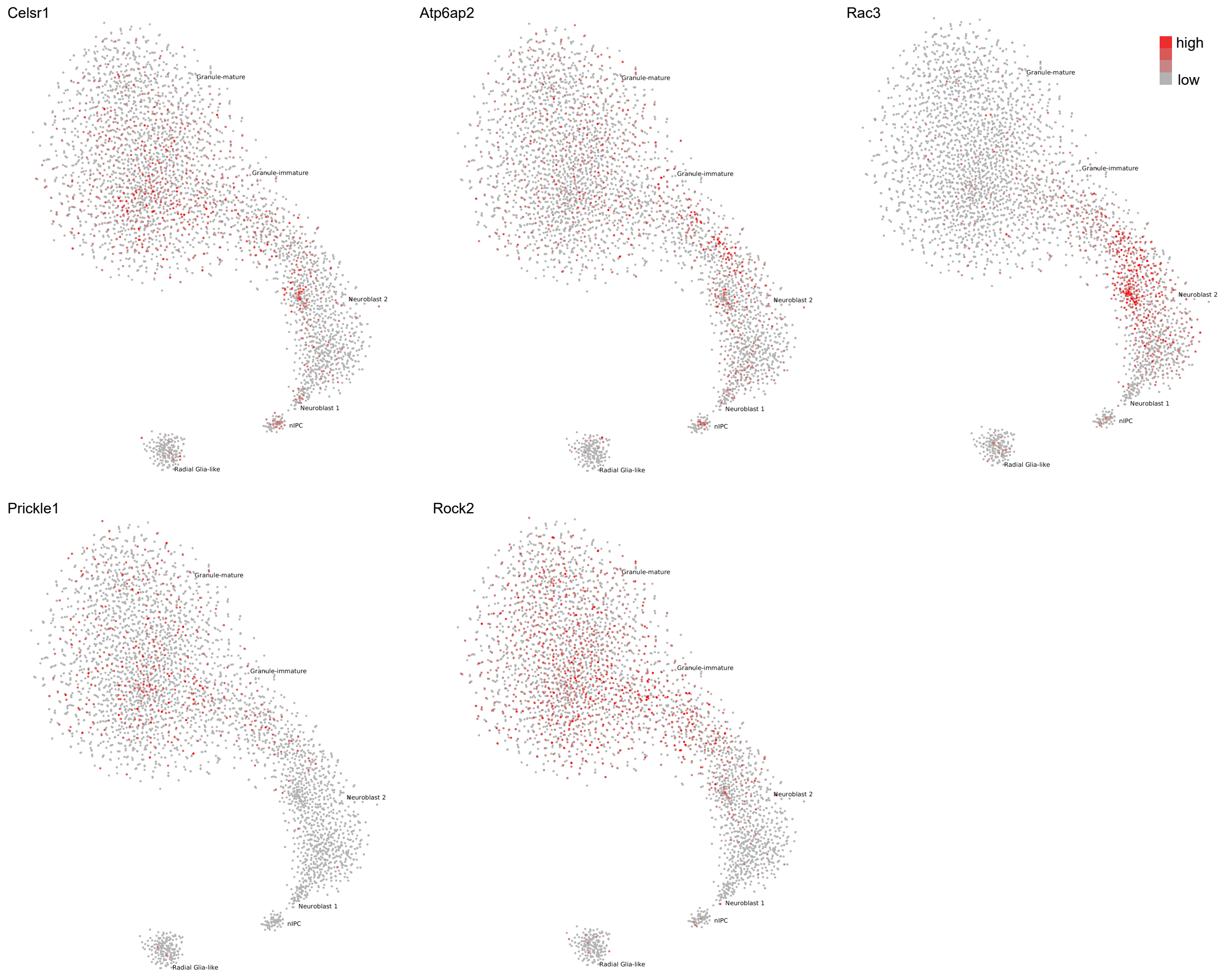

Wnt/Ca^{2+} signaling

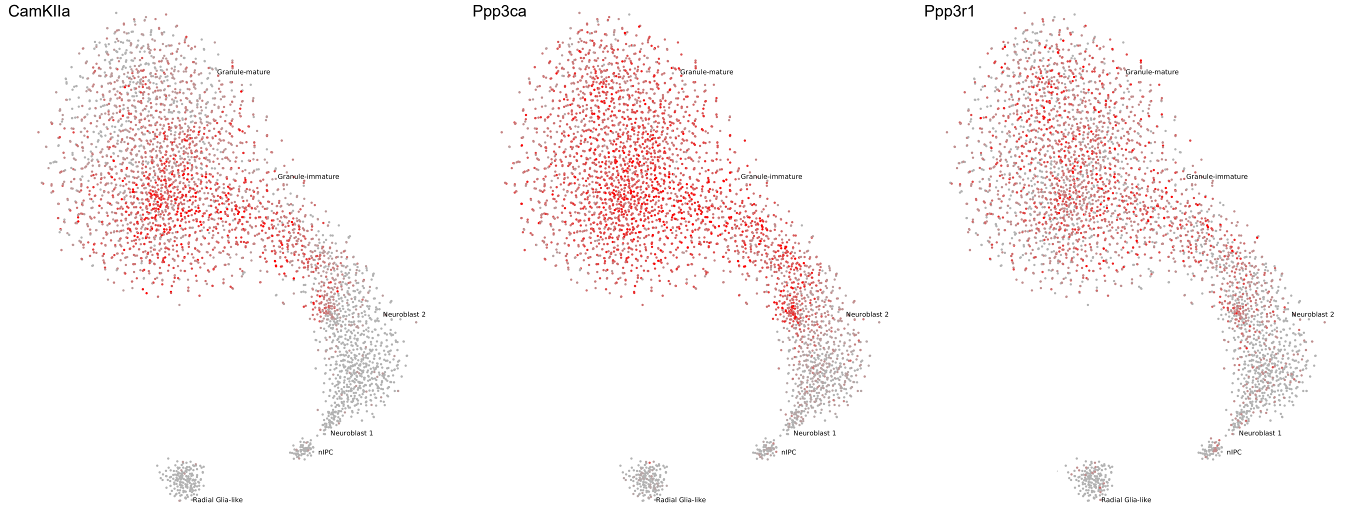

Wnt signaling ligands

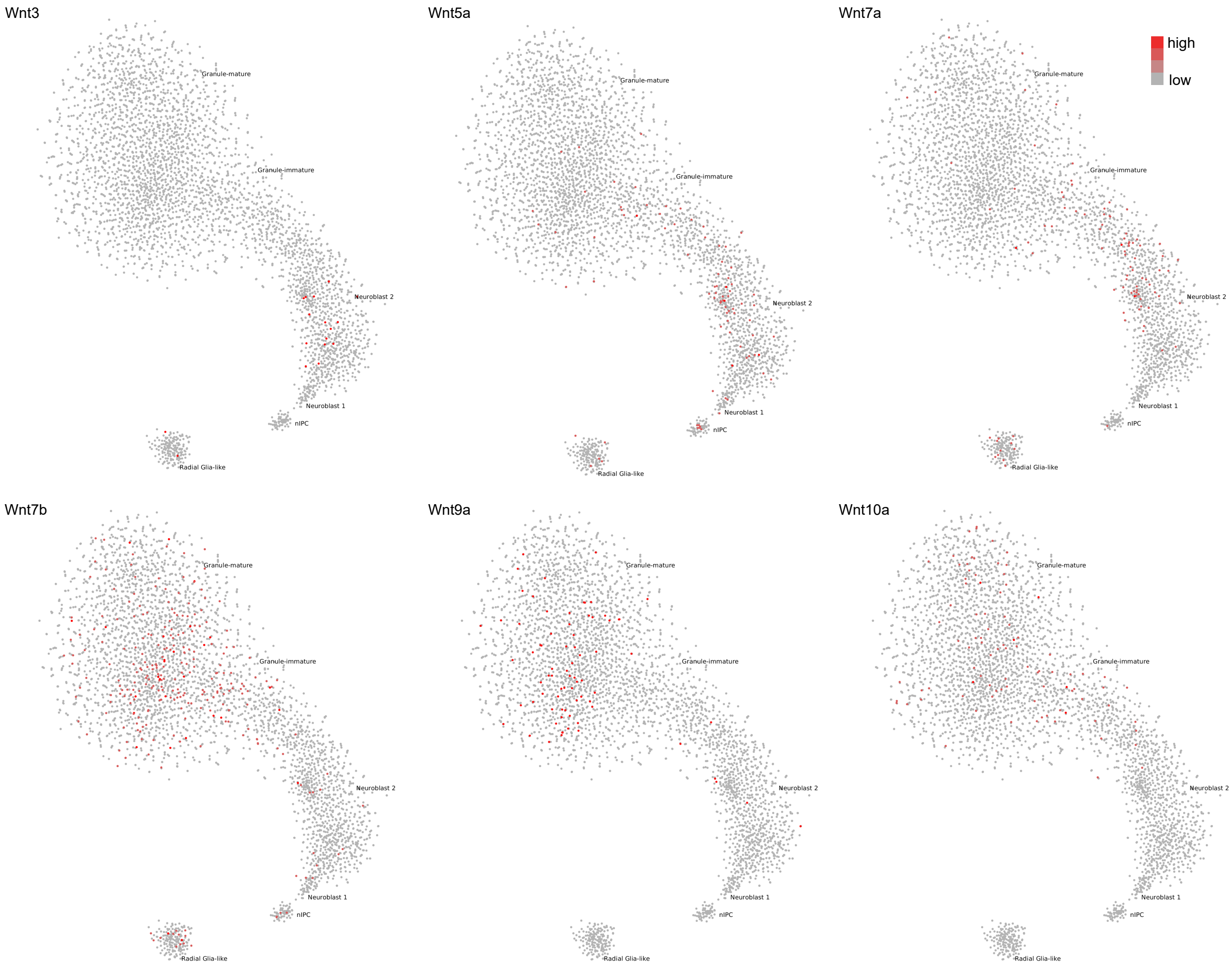

Wnt signaling inhibitors

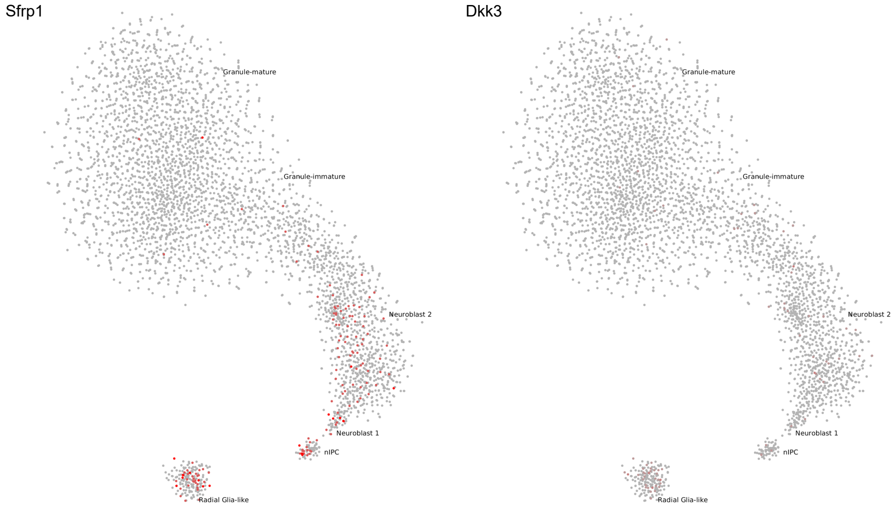

Wnt signaling receptors

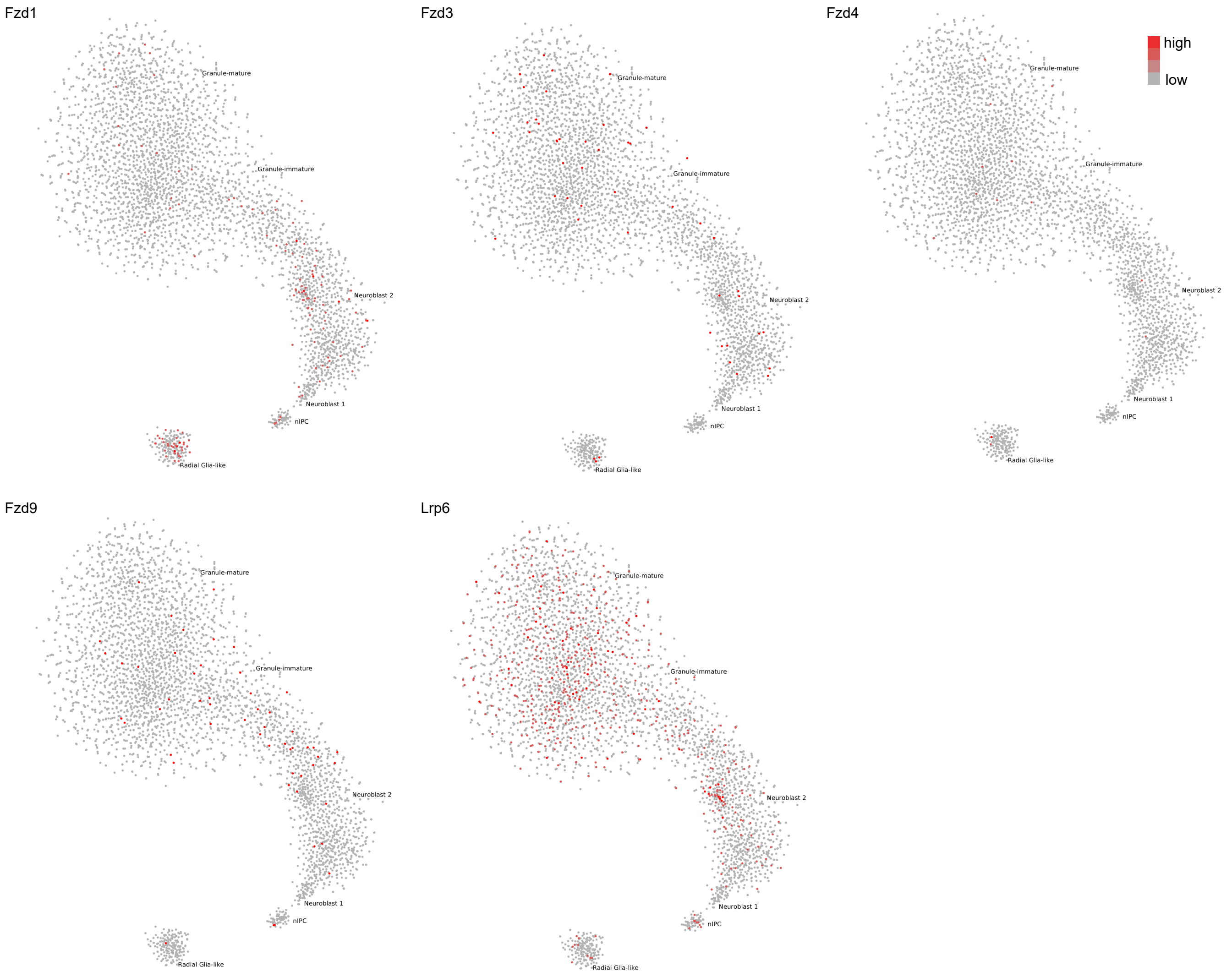

B

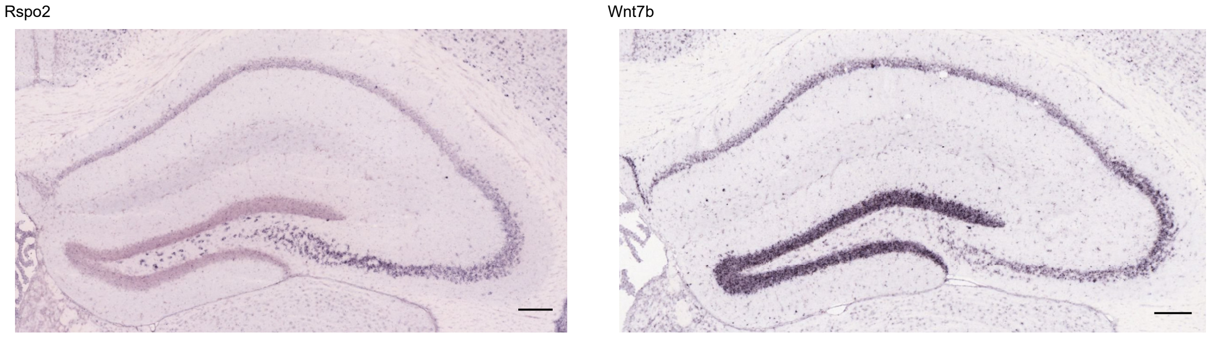

Appendix Fig. S1 Potential regulators of canonical Wnt signaling during adult neurogenesis

A tSNE visualizations of genes associated with Wnt/β-catenin signaling, Wnt/PCP signaling Wnt/Ca2+ signaling and Wnt signaling ligands, inhibitors and receptors that display a stage specific expression along the neurogenic lineage. Cells are stained for expression of consecutive genes: grey - low expression, red - high expression. Data set: single cell RNA-sequencing of 5454 cells of the murine DG of mice from Postnatal Day 12 to Postnatal Day 35. (Hochgerner et al, 2018)

B Representative images of in situ hybridizations of Rspo2 (Wnt signaling modulator; http://mouse.brain-map.org/gene/show/88569) and Wnt7b (Wnt ligand, http://mouse.brain-map.org/gene/show/22179) in the hippocampus of adult mice (P56) from the Allen brain atlas (© 2015 Allen Institute for Brain Science. Allen Brain Atlas API. Available from: brain-map.org/api/index.html). Scale bar= 200µm.
