# Supplementary material for: β‐catenin signaling modulates the tempo of dendritic growth of adult‐born hippocampal neurons
Source: EMBO J. 2020 Sep 15;39(21):e104472. doi: 10.15252/embj.2020104472 (PMC7604596; doi:10.15252/embj.2020104472)
Supplement: Supplementary file 2 — Expanded View Figures PDF [file EMBJ-39-e104472-s002.pdf]

Expanded View Figures

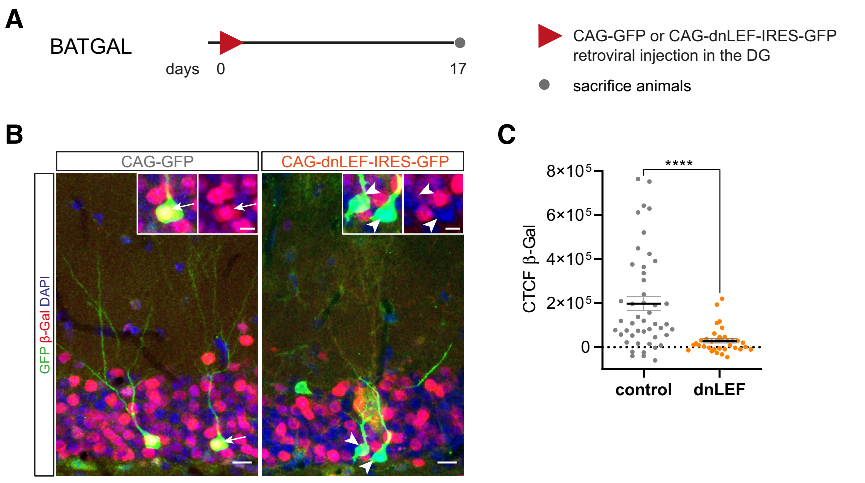

Figure EV1. Expression of dnLEF reduces canonical Wnt signaling activity in adult-born granule neurons.

A Experimental scheme of retroviral injection paradigm. Adult BATGAL mice were stereotactically injected with either the CAG-dnLEF-IRES-GFP (dnLEF) or CAG-GFP (control) MMLV and were sacrificed 17 dpi.

B Representative images of β-galactosidase reporter expression (red) in transduced neurons (green). Arrows and arrowheads point to transduced neurons with and without reporter signal, respectively. Scale bar = 10 μm. Insets show magnifications of transduced cells. Scale bar = 5 μm.

C Measurements of the corrected total cell fluorescence of the β-galactosidase signal in control and dnLEF neurons show reduced canonical Wnt signaling activity in neurons expressing dnLEF ($P < 0.0001$; control: $n = 47$ cells from three animals, dnLEF: $n = 35$ cells from three animals).

Data information: Data represented as mean ± SEM, significance was determined using two-tailed Mann–Whitney *U*-test, and significance levels are displayed in GP style (****$P < 0.0001$).

© 2020 The Authors

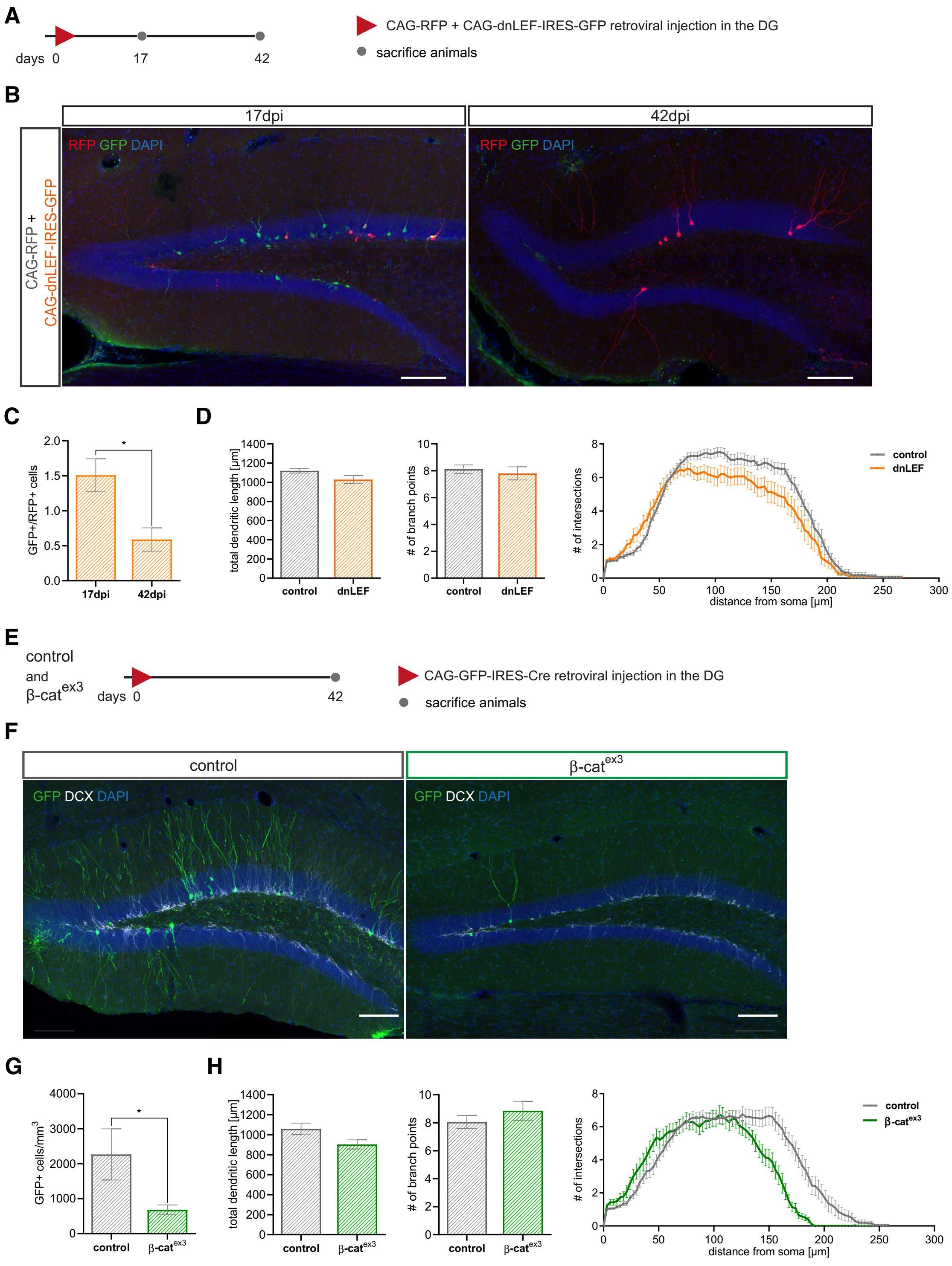

Figure EV2

 © 2020 The Authors

Figure EV2. Survival of neurons with loss and gain of β-catenin signaling is impaired.

- A Experimental scheme of retroviral injection paradigm. Adult mice were stereotactically co-injected with the CAG-dnLEF-IRES-GFP (dnLEF) and CAG-RFP (control) MMLVs and were sacrificed 17 and 42 dpi.
- B Representative images of the DG with transduced neurons at 17 and 42 dpi. CAG-dnLEF-IRES-GFP transduced cells in green, and CAG-RFP transduced cells in red. Scale bar = 100 µm.
- C Quantification of RFP-positive and GFP-positive cells show reduced survival of neurons transduced with dnLEF (P = 0.0286; n = 4 animals).
- D Morphology analysis of surviving neurons shows no difference in dendritic length (P = 0.1004) and number of branch points (P = 0.8654) between dnLEF and control neurons, but a reduction in complexity (Sholl analysis; P = 0.0176; control: n = 18 cells from four animals, dnLEF: n = 15 cells from four animals).
- E Schematic representation of the retroviral paradigm used to analyze survival of canonical Wnt signaling gain of function in neural progenitors.
- F Representative images of the dentate gyrus of control and β-cat^{ex3} mice with GFP expressing transduced neurons (green) and the immature marker doublecortin (DCX) (gray) at 42 dpi. Scale bar = 100 µm.
- G Quantification of GFP-positive cells in control and β-cat^{ex3} animals show reduced survival of neurons with stable expression of β-cat from the stage of fast-proliferating progenitor stage on (P = 0.0286; n = 4 animals).
- H Morphology analysis of surviving GFP-positive neurons shows no difference in dendritic length (P = 0.0503) and number of branch points (P = 0.5051), but differences in complexity in Sholl analysis (P = 0.0240) between genotypes (control: n = 20 cells from four animals, dnLEF: n = 15 cells from four animals).

Data information: Data represented as mean ± SEM, significance was determined using two-way ANOVA for Sholl analysis and two-tailed Mann–Whitney U-test for all other analyses, and significance levels were displayed in GP style (*P < 0.0332).

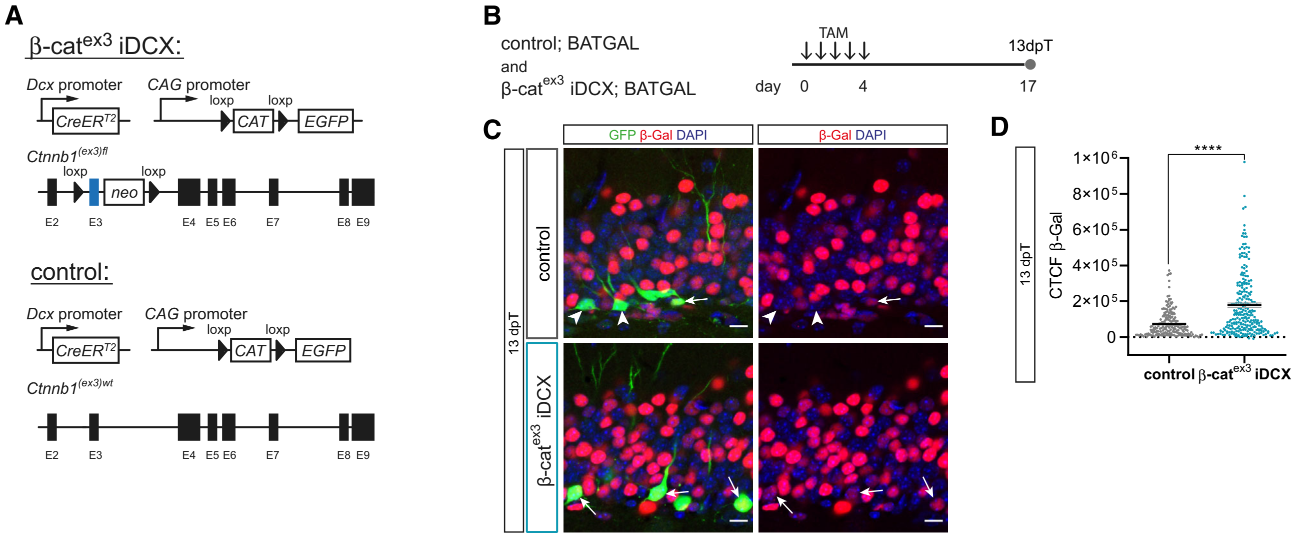

Figure EV3. Stabilization of β-catenin in β-cat^{ex3} iDCX mice leads to an increase in canonical Wnt signaling activity.

- A Schematic representation of the conditional alleles and transgenes of the β-cat^{ex3} iDCX mouse line and control iDCX mice.
- B To analyze the impact of β-catenin stabilization on canonical Wnt signaling, β-cat^{ex3} iDCX mice and controls were crossed with BATGAL mice. Tamoxifen (TAM) was applied i.p. every 12 h for 5 days. Animals were analyzed 13 days post-tamoxifen (dpT).
- C Representative images of β-galactosidase reporter expression (red) in GFP-positive cells (green) in control iDCX BATGAL and β-cat^{ex3} iDCX BATGAL mice. DAPI in blue. Arrows and arrowheads indicate β-galactosidase reporter-positive and negative cells, respectively. Note the prominent β-Gal signal intensity of surrounding non-recombined cells in the granule cell layer of the DG. Scale bar = 10 µm.
- D Measurements of corrected total cell fluorescence (CTCF) of the β-Gal signal in recombined cells at 13 dpT showed an increase in canonical Wnt signaling activity upon stabilization of β-catenin in β-cat^{ex3} iDCX BATGAL mice (P < 0.0001; control BATGAL: n = 205 cells from three animals, β-cat^{ex3} iDCX BATGAL: n = 248 cells from three animals).

Data information: Data represented as mean ± SEM, significance was determined using two-tailed Mann–Whitney U-test, and significance levels are displayed in GP style (****P < 0.0001).

© 2020 The Authors

© 2020 The Authors

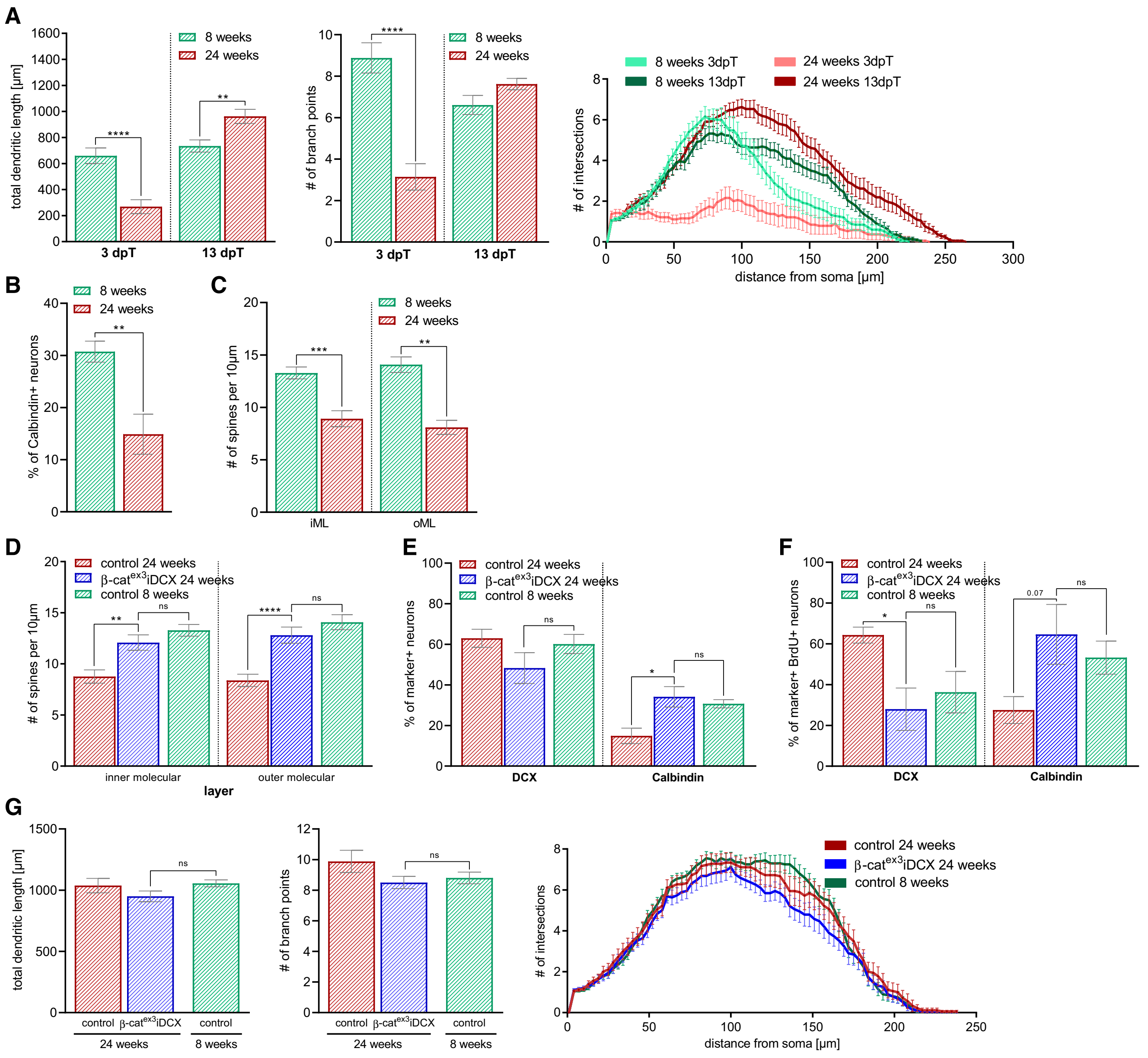

Figure EV4.

© 2020 The Authors

© 2020 The Authors

Figure EV4. Induction of β-catenin signaling activity in middle-aged mice rejuvenates dendrite and spine development.

- A Comparison of total dendritic length (3 dpT $P < 0.0001$, 13 dpT $P = 0.0028$), branch points (3 dpT $P < 0.0001$), and Sholl analysis (3 dpT $P = 0.0002$, 13 dpT $P = 0.0036$) between 8-week-old and 24-week-old control iDCX animals at 3 and 13 dpT showed age-associated delay in dendritic development (8 weeks 3 pT: control: $n = 25$ cells from nine animals, 8 weeks 13 dpT: control: $n = 23$ cells from nine animals, 24 weeks 3 dpT: control: $n = 21$ cells from five animals, 24 weeks 13 dpT: control: $n = 21$ cells from five animals). Data extracted from Figs 3C and D, and 5C.
- B Reduced percentage of recombined neurons expressing Calbindin in middle-aged control iDCX mice compared to young adult control iDCX mice 13 days after recombination ($P = 0.0020$; 8 weeks: $n = 10$ animals, 24 weeks: $n = 8$ animals). Data extracted from Figs 3G and 6F.
- C Comparison of spine number in 8-week-old and 24-week-old control iDCX mice showed reduced spine density in the inner and outer molecular layer of the dentate gyrus at 13 dpT (iML $P = 0.0009$, oML $P = 0.0023$; 8 weeks: $n = 20$ cells from five animals, 24 weeks: $n = 19$ cells from four animals). Data extracted from Figs 3E and 6D.
- D Analysis of spine density in 24-week-old control and β-cat^{ex3}iDCX in comparison with 8-week-old control mice. In the inner molecular and outer molecular layer of the DG at 13 dpT. The number of spines in 24 weeks old resembled the number of spines in 8-week-old control mice (iML $P = 0.2211$, oML $P = 0.0810$), indicating a rescue of delayed spinogenesis in middle-aged animals (24 weeks: control $n = 19$ cells from four animals, β-catex3 iDCX: $n = 20$ cells from four animals, 8 weeks: control: $n = 20$ cells from five animals). Data extracted from Figs 3E and 6D.
- E The fraction of neurons with DCX and Calbindin expression in 24-week-old β-cat^{ex3}iDCX animals at 13 dpT was comparable to the fraction in 8-week-old control animals (DCX $P = 0.1593$, Calbindin $P = 0.6842$; 24 weeks: control: $n = 8$ animals, β-cat^{ex3} iDCX: $n = 10$ animals, 8 weeks: $n = 10$ animals). Data extracted from Figs 3G and 6F.
- F Birthdated neurons in 24-week-old β-cat^{ex3}iDCX animals and 8-week-old control animals showed a comparable fraction of neurons expressing DCX ($P = 0.9714$) and Calbindin ($P = 0.8968$) at 13 dpT (24 weeks: control: $n = 5$ animals, β-cat^{ex3} iDCX: $n = 5$ animals, 8 weeks: control: $n = 5$ animals). Data extracted from Figs 4B and 7C.
- G Birthdated neurons in 24-week-old control, β-cat^{ex3}iDCX and 8-week-old control animals have comparable dendritic parameters (total dendritic length [$P = 0.0718$], number of branch points [$P = 0.7235$], and Sholl analysis [$P = 0.0967$]) at 13 dpT (24 weeks: control: $n = 18$ cells from four animals, β-cat^{ex3} iDCX: $n = 20$ cells from four animals, 8 weeks: control: $n = 20$ cells from four animals). Data extracted from Figs 4F and 7G.

Data information: Data represented as mean ± SEM, significance was determined using two-way ANOVA for Sholl analysis and two-tailed Mann–Whitney *U*-test for all other analyses, and significance levels are displayed in GP style (*$P < 0.0332$, **$P < 0.0021$ and ***$P < 0.0002$, ****$P < 0.0001$).

© 2020 The Authors
